# An Improved and Simplified *Agrobacterium*-Mediated Genetic Transformation Protocol for *Solanum nigrum* with a Shorter Growth Time

**DOI:** 10.3390/plants13152015

**Published:** 2024-07-23

**Authors:** Qianqian Li, Xiuyuan Wang, Chong Teng, Xuxia He, Xinyue Fu, Wentao Peng, Yinglun Fan, Shanhua Lyu

**Affiliations:** College of Agriculture, Liaocheng University, Liaocheng 252000, China; 2110190208@stu.lcu.edu.cn (Q.L.); 171497553@foxmail.com (X.W.);

**Keywords:** *Solanum nigrum*, speeding up breeding, *Agrobacterium*-mediated genetic transformation, tissue culture, induction of rooting, *Dianthus caryophyllus*

## Abstract

*Solanum nigrum* (Solanaceae family) is widely consumed as a fruit or local leafy vegetable after boiling; it also serves as a medicinal plant. Although *Agrobacterium*-mediated genetic transformation has been established in *S*. *nigrum*, the transformation period is long. Specifically, induction of roots takes approximately five weeks for tetraploid and hexaploid *S*. *nigrum*, and 7 weeks for diploid *Solanum americanum*. In this study, we developed an improved rooting-induced method that requires only about 1 week and avoids the use of tissue culture. After generating the transgenic shoots, they were directly transplanted into the soil to facilitate root formation. Remarkably, 100% of the transgenic shoots developed roots within 6 days. Our improved method is time-saving (saving more than 1 month) and simpler to operate. The improved rooting-induced step can be applied to induce roots in various plants using tissue culture, exemplified by the carnation (*Dianthus caryophyllus* L.). Furthermore, we applied the improved method to generate *S*. *americanum* plants expressing *AcMYB110* from kiwifruit (*Actinidia chinensis* spp.). This method will contribute to speeding up gene functional analysis and trait improvement in *S*. *nigrum* and might have potential in fast plant molecular breeding processes in crops and rapid rooting induction in tissue culture.

## 1. Introduction

*Solanum nigrum*, an annual herb belonging to the eudicots group, is part of the Solanaceae family. It includes several species such as the diploid *Solanum americanum* (2n = 2x = 24), the hexaploid *S*. *nigrum* L. (commonly known as black nightshade, 2n = 6x = 72), and its variety, the tetraploid *S*. *nigrum* L. var. suaveolens (yellow nightshade, 2n = 4x = 48). *S*. *nigrum* is globally distributed and consumed locally as a fruit or green leafy vegetable in some regions. The toxic glycoalkaloid solanine in *S*. *nigrum* can be degraded into a non-toxic substance by boiling or cooking. The mature fruit is particularly valued for its high nutritional content [1,2,3]. The entire *S*. *nigrum* plant is traditionally used in Chinese medicine for its varied therapeutic properties, which include promoting blood circulation, reducing swelling, fever reduction, liver protection, improving glucose tolerance, and reducing body weight and body fat [3,4,5,6,7,8]. Additionally, *S*. *nigrum* is a known hyperaccumulator of metals and is used extensively in the study of remediation for soils contaminated with heavy metals [7,9]. *S*. *nigrum* also serves as a model plant in ecological studies to explore plant–herbivore interactions and the associated signaling pathways [10,11]. Enhancing desirable traits in *S*. *nigrum*, such as improving the nutritional value and quality of its vegetables and fruits, understanding and optimizing the synthetic pathways of its medicinal compounds, and investigating heavy metal uptake and accumulation, relies heavily on genetic transformation techniques. These approaches are crucial for manipulating target genes in medicinal compound synthesis and understanding complex ecological interactions.

The *Agrobacterium*-mediated genetic transformation method was previously established in *S*. *nigrum*, utilizing a sterile rooting culture medium for inducing roots in transgenic shoots [2,3]. This process typically lasted about 5 weeks for tetraploid and hexaploid *S*. *nigrum*, and 7 weeks for diploid *S*. *americanum*. This duration was considerably lengthy. Additionally, transplanted whole transgenic plantlets often suffered from labor-intensive removal of agar and damaged root hairs. In our study, we developed an improved and convenient transformation method that significantly shortens the transformation period, allowing for the generation of transgenic *S*. *nigrum* plantlets capable of soil growth. This method facilitates root induction directly in soil, a non-sterile environment. This new approach simplifies the generation of soil-growth plantlets and represents a significant advancement in genetic transformation techniques. We believe this method can be broadly applied to enhance genetic transformation protocols and expedite tissue culture propagation in other plant species as well.

## 2. Materials and Methods

### 2.1. Plant Materials and Growth Conditions

Seeds of *S*. *nigrum* (including diploid *S*. *americanum*, tetraploid *S*. *nigrum* L. var. Suaveolen, hexaploid *S*. *nigrum* L.) and carnation (*Dianthus caryophyllus*) were sourced from a local market. The plants were cultivated in a greenhouse at 22 ± 2 °C under a 16 h light/8 h dark photoperiod.

### 2.2. Agrobacterium Tumefaciens-Mediated Genetic Transformation and Generation of Genetically Modified S. nigrum Plantlets Grown in Soil

**Figure 1:**
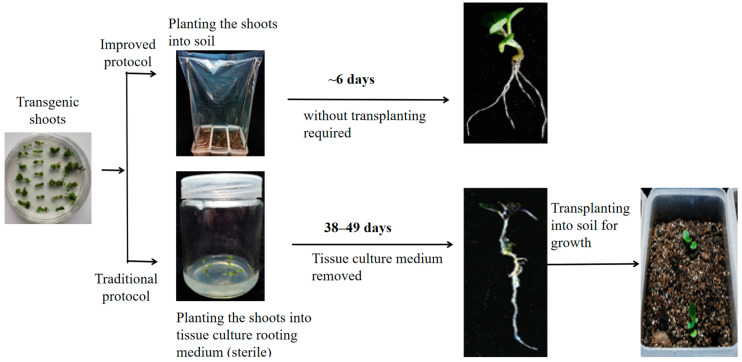
Diagram of the improved *Agrobacterium*-mediated genetic transformation protocol for *S. nigrum* compared with the traditional method.

In the traditional transformation protocol for *S*. *nigrum*, the transgenic plantlets were generated following previously described methods [3]. Initially, *S*. *nigrum* seeds were sterilized using 70% (*v*/*v*) ethanol for 1 min followed by a 3% (*w*/*v*) sodium hypochlorite solution for 15 min, then rinsed five times with sterile water. These sterilized seeds were then placed on half-strength Murashige and Skoog (MS) agar plates (consisting of 1/2 MS (2.215 g/L) + 2% sucrose + 0.7% agar) and cultured in a greenhouse for 10 days. The MS basal medium (with vitamins) and agar were bought from Phyto-Tech Labs (Lenexa, Kansas, USA) and Solarbio (Beijing, China), respectively. The *A*. *tumefaciens* strain LBA4404, transformed with the plant binary expression vector pR35BTR1 carrying the *DsRed2* reporter gene under the control of the CaMV 35S promoter was used to infect the explants [12]. The pR35BTR1 was transformed into the LBA4404 by electroporation. The *A*. *tumefaciens* cells (stored in −80 °C; Luria-Bertani broth containing 10% glycerol stock) were streaked on the LB plates with 50 µg/mL kanamycin and 20 µg/mL rifampicin and incubated at 28 °C for 2 days. Subsequently, a single colony was cultured in 10 mL of liquid LB medium overnight at 28 °C with shaking at 200 rpm. The next day, 1 mL of this culture was transferred to 100 mL of fresh liquid LB medium and further cultured at 28 °C for approximately 7 h with shaking at 200 rpm until the OD600 reached 0.6. The freshly prepared *A*. *tumefaciens* cells were then centrifuged at 4000 rpm for 10 min, gently resuspended in liquid MS media (4.43 g/L) supplemented with 2% sucrose and 0.1 mM acetosyringone (MS induction medium, MSIn), and used to infect the explants. Seedlings aged 10 days were dissected into small segments (0.5–1 cm) and co-incubated for 20 min with the *A*. *tumefaciens* cultures carrying the pR35BTR1 binary construct [12]. After removing excess *A*. *tumefaciens* culture using sterilized filter papers, the infected explants were transferred to MSIn media with 0.7% agar and cultured at 19–20 °C in darkness for 2 days. Following this period, the infected explants were washed five times with MS liquid media containing 500 mg/L cefotaxime and then transferred to MS selection and differentiation culture media (MS selection and differentiation media, MSSD, consisting of 4.43 g/L MS, 1 mg/L Zeatin, 400 mg/L cefotaxime, 2% sucrose, and 0.7% agar). The calli or buds were transferred to the same fresh culture medium at three-week intervals. Once the shoots reached approximately 2 cm in length, the regenerated plantlets were moved to MS rooting medium (consisting of 4.43 g/L MS, 1 mg/L NAA, 2% sucrose, and 0.7% agar). The traditional transformation method served as a control. Our improved protocol involves the rooting-induced step, where the elongated transgenic shoots are cut and directly planted into pots containing a mixture of nutrient soil and vermiculite (1:2 ratio). These are then cultivated in a greenhouse. Initially, the pots are covered with a transparent polyethylene plastic bag for 4–5 days. Over the next 2–4 days, ventilation holes are gradually made in the bag to help the transgenic plantlets acclimate to greenhouse conditions. Subsequently, the plastic bag is removed, and cultivation continues in the greenhouse. The transformation approach is illustrated in Figure 1.

### 2.3. In Vitro Tissue Culture Regeneration of Carnation

The carnation shoot regeneration was produced following the protocols for the generation of genetically modified *S*. *nigrum* plantlets in this study, with minor modifications. The hypocotyls were used as explants from 16-day-old seedlings. The explants were not infected with *A*. *tumefaciens*.

### 2.4. 35S::AcMYB110 Vector Construction and Transformation into the Diploid S. americanum

An R2R3-MYB transcription factor, the full length of kiwifruit (*Actinidia chinensis*) *AcMYB110* (GenBank no. KF311107) was cloned into the binary expression vector pBI121 (BD Biosciences Clontech), resulting in *AcMYB110* being expressed under the CaMV35S promoter [3]. The *35S*::*AcMYB110* binary expression construct was transformed into the *A*. *tumefaciens* strain LBA4404, producing *35S*::*AcMYB110* transgenic *S*. *americanum* plantlets using our modified transformation method.

### 2.5. PCR (Polymerase Chain Reaction), Semi-Quantitative RT-PCR Analysis, and Fluorescent Protein Observation

To identify the transgenic plants, PCR and semi-quantitative RT-PCR were conducted as described in papers published previously [3,13]. The DsRed2 fluorescent protein in transgenic lines was detected using the Tanon-5200Multi machine (Tanon Co., Ltd., Shanghai, China) with an excitation wavelength of 540 nm and emission wavelength of 600 nm.

### 2.6. Root Morphological Parameters

At 6 dpt (days post-transplanting; the day that the elongated shoot was transplanted to the soil was designed as day 0), 38 dpt (for tetraploid and hexaploid *S*. *nigrum*), and 49 dpt (for diploid *S*. *americanum*), root samples were washed with water and the growth medium was removed with forceps. Root length and morphological indices analyses were conducted as previously described [14]. An EPSON V700 Scanner (EPSON (China) Co., Ltd., Beijing, China) and the WinRHIZO PRO 2012 Root Analysis System (Regent Instruments Inc., Quebec, QC, Canada) were used to capture the image and analyze the image, respectively.

### 2.7. Statistical Analysis

Microsoft Office Excel 2021 and DPS v9.50 standard (Data Processing System) statistical software were used for data analysis. We calculated the means ± standard deviations of three independent experiments. The dry weights of transgenic plantlets derived from elongated shoots and roots were measured following a specified method [14]. The dry weight was determined by heating at 105 °C for 20 min and then at 80 °C for 12 h. In each independent experiment, 13 distinct transgenic lines were analyzed.

## 3. Results

### 3.1. Establishment of an Improved Genetic Transformation Method for Generating Transgenic S. nigrum Plantlets Grown in Soil

**Figure 2:**
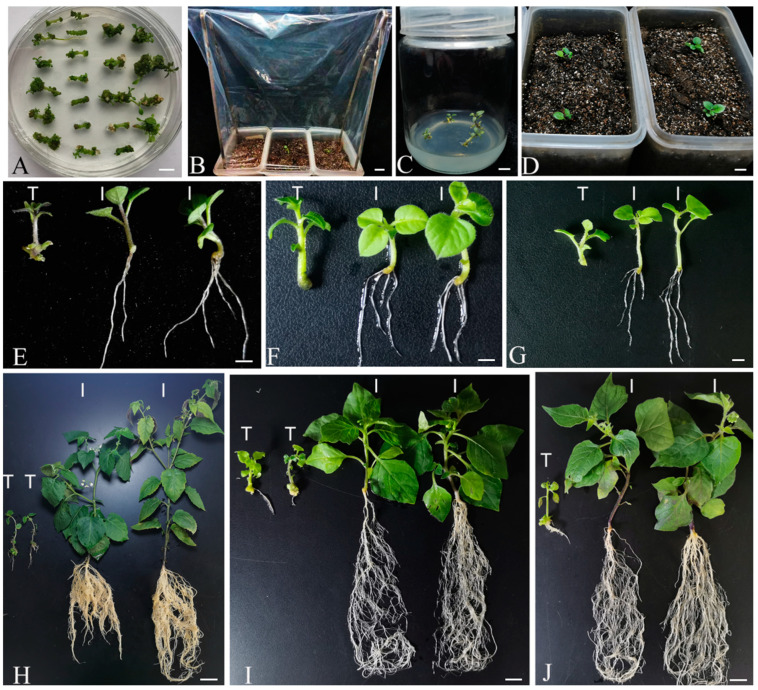
The improved *Agrobacterium*-mediated genetic transformation protocol for *S*. *nigrum*. The improved genetic transformation protocol for diploid *S. americanum* (**A**–**E**,**H**), tetraploid *S. nigrum* (**F**,**I**), and hexaploid *S. nigrum* (**G**,**J**). Induction of transgenic shoots (**A**). Transgenic shoots were covered with a plastic bag for 4–5 d for acclimation to the environment after transplanting in the soil (**B**). As a control, transgenic shoots were transplanted in the rooting medium in the traditional transformation method at 6 dpt (**C**). The transgenic grown soil plantlets produced via the improved transformation method at 6 dpt (**D**). A comparison of the transgenic plantlets between our improved transformation method and the traditional method. The transgenic plantlets were grown at 6 dpt (**E**–**G**), 49 dpt (**H**), 38 dpt (**I**), and 38 dpt (**J**). Letters T and I indicate transgenic plant(s) produced via the traditional transformation method and improved transformation method, respectively. Bars = 1 cm.

**Figure 3:**
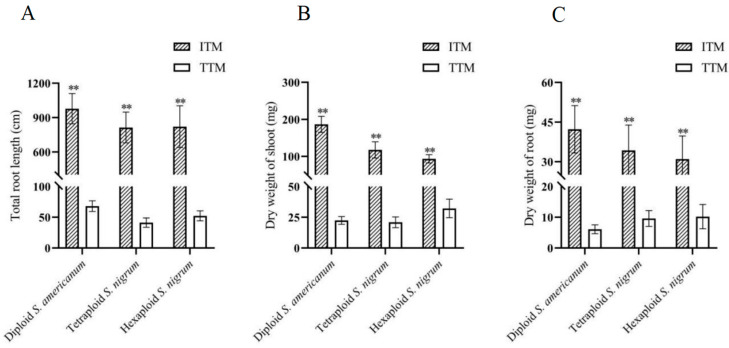
A comparative analysis of the improved method and traditional transformation methods in three *S. nigrum* species. A comparative anslysis of total root lengths (**A**), dry root weights (**B**), and dry shoot weights (**C**). The data were measured at 49 dpt (diploid *S. americanum*), 38 dpt (tetraploid *S. nigrum*), and 38 dpt (tetraploid *S. nigrum*). ** *p* < 0.01. Values are mean ± SD of three independent replicates. ITM: improved transformation method; TTM: traditional transformation method.

In our study, we developed an improved transformation protocol for *S*. *nigrum*. The transgenic shoots, approximately 2 cm in length (Figure 2A), were directly planted in the moist nutrient soil mixed with vermiculite (1:2) (Figure 2B) and grown in a greenhouse throughout their life cycle. At the beginning of transplanting, transgenic shoots were covered with a transparent plastic bag to maintain high humidity for 4–5 days, aiding their adaptation to the growing conditions (Figure 2B). As a control, regenerated shoots were transferred to a rooting medium using a traditional method detailed in the materials and methods section. Figure 2C,D show the transgenic plantlets grown in the tissue culture rooting medium and soil, respectively, at 6 days post-transplant (dpt). At 6 dpt, the growth medium—either agar or soil—was carefully removed from around the transgenic shoots/plantlets. The plantlets are displayed in Figure 2E (diploid), Figure 2F (tetraploid), and Figure 2G (hexaploid *S. nigrum*). Using our improved protocol, 100% of the tested transgenic shoots of diploid, tetraploid, and hexaploid *S*. *nigrum* developed roots by 6 dpt, with total root lengths of 29 ± 7 cm, 26 ± 2 cm, and 29 ± 5 cm, respectively (Figure 2E–G). In contrast, using the traditional method, no roots were produced by the transgenic shoots at 6 dpt (Figure 2E–G). Up to 49 dpt for diploids and 38 dpt for tetraploid *S. nigrum* and hexaploid *S. nigrum*, the total root lengths for plantlets grown using the traditional method were 68 ± 9 cm for diploids, 41 ± 8 cm for tetraploids, and 52 ± 8 cm for hexaploids. The respective dry root weights were 6 ± 1 mg, 10 ± 3 mg, and 10 ± 4 mg, while the shoot weights were 22 ± 3 mg, 23 ± 4 mg, and 32 ± 8 mg (Figure 2H–J and Figure 3). By contrast, in our improved method, the transgenic plantlets exhibited significantly faster growth than those produced by the traditional method with blooming observed (Figure 2H–J). The total root lengths, dry root weights, and dry shoot weights of transgenic plants in the improved method reached 978 ± 132 cm, 42 ± 9 mg, and 187 ± 22 mg for diploids, respectively (Figure 2H and Figure 3A–C); 814 ± 134 cm, 34 ± 10 mg, and 117 ± 22 mg for tetraploids, respectively (Figure 2I and Figure 3A–C); and 821 ± 182 cm, 31 ± 9 mg, and 93 ± 11 mg for hexaploids, respectively (Figure 2J and Figure 3C). This improved method reduced the time required for rooting by 6 weeks in diploids, and by about 4 weeks in tetraploid and hexaploid *S. nigrum*, leading to an earlier fruit ripening period of about 4–6 weeks compared to the traditional method.

**Figure 4:**
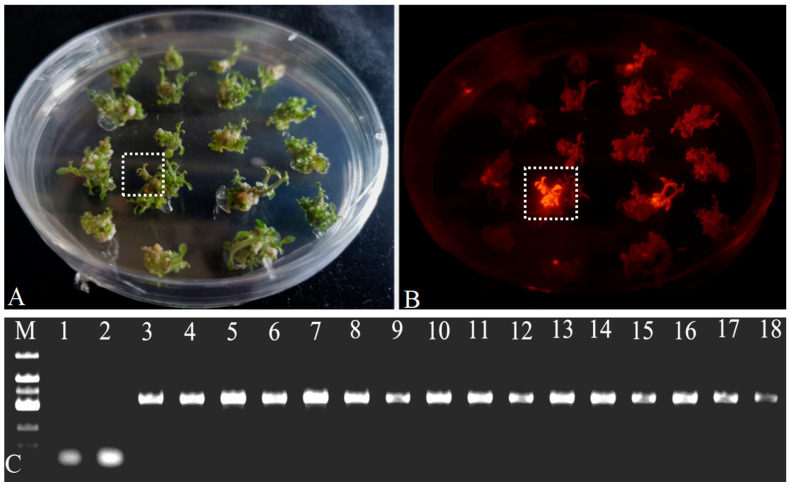
Observation of red fluorescence in *DsRed2*-transgenic *S. americanum* shoots. Shoots-induced were photographed under bright light (**A**) and excitation wavelength at 540 nm/emission wavelength at 600 nm, respectively (**B**). Strong fluorescence of DsRed2 was observed in the transgenic shoots-induced (shown in the rectangle box). Bars = 1 cm. PCR analysis of *DsRed2* gene (678 bp) in *S. americanum* (**C**). Lane 1, ddH_2_O used as a template (blank control); Lane 2, Wild-type plant used as a negative control; Lane 3, pR35BTR1 plasmid used as a template (positive control); Lane 4–18, *DsRed2*-positive independent transgenic plant transformed with pR35BTR1 plasmid; M, DL2000 bp DNA ladder (Sangon Biotech Co., Shanghai, China).

The transgenic plantlets were confirmed through the expression of DsRed2 protein, PCR, and RT-PCR analyses. The presence of visible fluorescence from DsRed2 corresponded to positive PCR results and the expression of *DsRed2* was detected by RT-PCR, confirming the consistency and reliability of the genetic modifications (Figure 4).

### 3.2. The Improved Genetic Transformation Method Was Used for Rapid Induction of Rooting in Carnation

**Figure 5:**
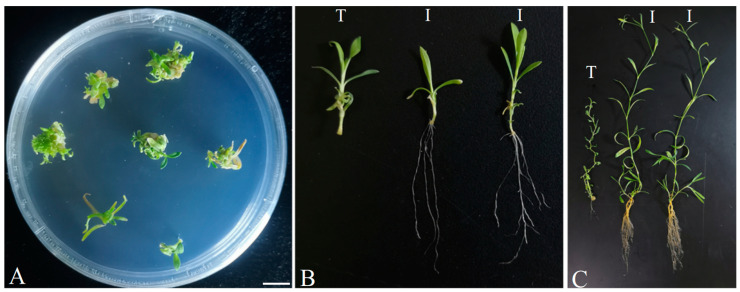
Rapid root generation in carnations using our improved method. Induction of shoots (**A**). Comparison of the transgenic plantlets generated using the improved method and the traditional method (**B**,**C**). The transgenic plantlets were grown at 10 dpt (**B**) and 75 dpt (**C**), respectively. Letters T and I indicate transgenic plant(s) produced via the traditional transformation method and the improved transformation method, respectively. Bars = 1 cm.

Carnations are one of the most important cut flowers worldwide. In vitro propagation is the most frequently and widely used propagation method for carnation breeding [15]. To test whether the improved method is suitable for rapid propagation depending on tissue culture in the induction of rooting, carnations were analyzed. The results indicated that the improved method is applicable to carnations (Figure 5).

### 3.3. Application of the Improved Genetic Transformation Method to Analyze the Overexpression of the Kiwifruit AcMYB110 in S. americanum

**Figure 6:**
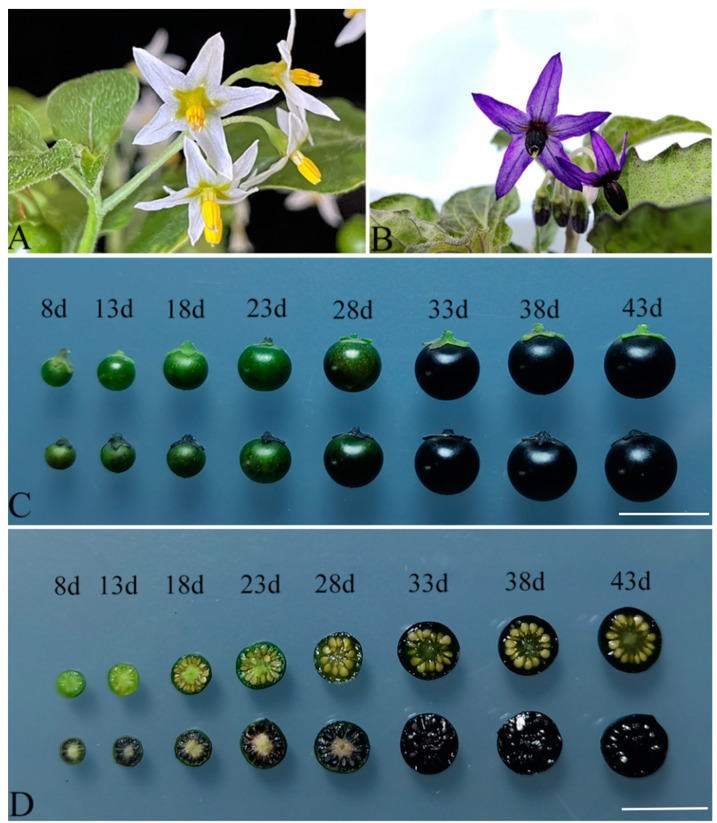
Ectopic expression of *AcMYB110* in *S. americanum* plants induced anthocyanin accumulation in the flowers and fruits. Wild-type flowers (**A**); flowers of *35S::AcMYB110* overexpression plant (**B**); fruits at different developmental stages; wild-type fruits (upper row) and *35S::AcMYB110* fruits (lower row) (**C**,**D**); section D shows cross-sections of the fruits. Bars = 0.5 cm.

**Figure 7:**
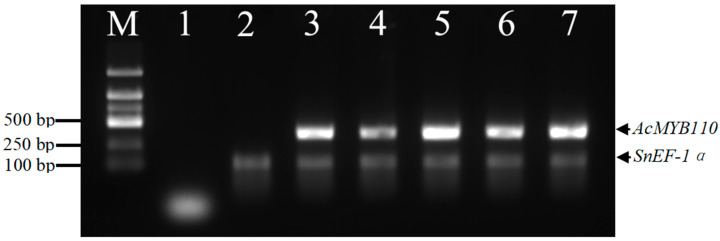
RT-PCR analysis of transgenic *S. americanum* expressing *AcMYB110*. Lane 1, ddH_2_O used as a template (blank control); Lane 2, Wild-type plant used as a negative control; Lanes 3–7, *DsRed2*-positive independent transgenic plants transformed with *pBM110* plasmid; M, DL2000 bp DNA ladder (Sangon Biotech Co., Shanghai, China). SnEF-1α was used as the reference gene.

In this study, we evaluated our improved method by analyzing the overexpression of kiwifruit *AcMYB110* in diploid *S*. *americanum*. We obtained twenty-five independent transgenic lines. The *35S::AcMYB110* transgenic plants exhibited high levels of anthocyanin accumulation in both flowers and fruits (Figure 6). Overexpression of *AcMYB110* in *S*. *americanum* was confirmed by semi-quantitative RT-PCR (Figure 7). The levels of anthocyanin accumulation correlated with *AcMYB110* transcript levels in various lines (Figure 7). We selected transgenic lines with pronounced phenotypic alterations for further analysis. The petals and anthers of the *35S::AcMYB110* overexpression plants turned purple, unlike the white color observed in wild-type lines (Figure 6A,B). In the fruit, anthocyanins accumulated throughout all stages of fruit development. In the mature fruits, both the fruit pulp and seeds exhibited a black color due to high anthocyanin accumulation (Figure 6C,D).

## 4. Discussion

### 4.1. A Simple and Rapid A. tumefaciens-Mediated Genetic Transformation Protocol Accelerates the S. nigrum Molecular Breeding Process

An efficient *Agrobacterium tumefaciens*-mediated genetic transformation method has previously been detailed for hexaploid *S*. *nigrum* [3,16]. The traditional method required about 5 weeks to induce root generation in tissue culture. By contrast, our newly developed protocol induced root generation in just six days in the hexaploid *S. nigrum*. We also applied the improved genetic transformation protocol to diploid and tetraploid *S. nigrum*. The results showed that our improved method can reduce the time required to generate transgenic plantlets by approximately 6 weeks in diploid *S. americanum* and 4 weeks in both tetraploid and hexaploid *S. nigrum* forms. Therefore, we established a simplified and rapid *A*. *tumefaciens*-mediated genetic transformation protocol for *S. nigrum*, significantly shortening the transformation period and accelerating the molecular breeding process of *S. nigrum*.

Additionally, the improved method is applicable to rapid propagation through tissue culture in carnations. Based on these findings, we conclude that our improved method could be broadly applied to optimize genetic transformation protocols and facilitate rapid propagation via tissue culture conditions in other plant species besides *S. nigrum*. This transformation procedure is a promising strategy for shortening the genetic transformation period, which relies on tissue culture for functional gene characterization and trait optimization, such as genome-editing-based crop improvement.

In the previous transformation method for hexaploid *S. nigrum*, rooting induction was conducted in a sterile tissue culture medium. Prolonged tissue culture time increased the risk of contamination. After root development, the entire transgenic plantlets had to be transferred to a new pot with nutrient soil [3,16]. The roots, which penetrated deeply into the agar media, were difficult and labor-intensive to extract without damaging the roots and root hairs. Our improved protocol allows transgenic shoots to grow directly in nutrient soil, eliminating the tedious step of detaching roots from the agar medium. The method does not require sterile conditions for rooting induction. Rooting induction and plantlet growth occur in the same growth media, a mixture of nutrient soil and vermiculite (1:2 ratio). Thus, our improved approach is time-efficient, labor-saving, convenient, highly efficient, and may reduce the risk of contamination.

Our improved method reduces the genetic transformation time by approximately 4–6 weeks, depending on the *S. nigrum* species. The rapid rooting of the transgenic shoots in our protocol is likely due to the dark soil environment, as darkness sufficiently promotes adventitious root formation [17].

### 4.2. The Conserved but Slightly Divergent Function of AcMYB110 in Regulating Anthocyanin Biosynthesis in Flowers and Fruits of S. nigrum Species

Previous studies have shown that overexpressing *AcMYB110* in plants leads to the accumulation of red-colored anthocyanins in both the petals and all regions of the fruit flesh and skin in *A. chinensis* and hexaploid *S. nigrum* [18,19]. This study finds that ectopic expression of *AcMYB110* in diploid plants results in purple anthocyanin accumulation in the flowers and fruits throughout all developmental stages (Figure 6). The phenotypes observed in *35S*::*AcMYB110* diploid *S. americanum* plants were similar to those in *A. chinensis* and hexaploid *S. nigrum*, indicating a high level of anthocyanin accumulation in both flowers and fruits. Previous studies have also shown that certain R2R3-MYB transcription factors can independently activate anthocyanin biosynthesis, such as the ectopic expression of *Arabidopsis AtMYB75*/*PAP1* in various plants including soybean, *Lotus japonicus*, *Medicago truncatula*, and tomato roots [13,20], and tobacco *MYBA* in grape hyacinth (*Muscari armeniacum*) [21]. These findings suggest that *AcMYB110* may positively regulate and independently activate anthocyanin biosynthesis in a broad range of plant species.

It is important to note that differences were observed between the diploid *35S::AcMYB110 S. americanum* plants, which accumulated purple-colored anthocyanins, and the hexaploid transgenic plants, which accumulated red-colored anthocyanins [19]. The composition and concentration of anthocyanins contribute to a wide range of colors, including orange, red, purple, and blue, in various plant organs [22]. The anthocyanin biosynthesis pathway is complex and regulated by multiple transcription factors, a small RNA family, and related genes coding for enzymes involved in anthocyanin biosynthesis at multiple levels. Consequently, the diverse species of anthocyanins found throughout the plant kingdom produce a spectrum of colors in flowers [23,24,25]. Over time, it is likely that sequence variations in the key enzymes of anthocyanin synthesis, which are recognized and bound by *AcMYB110*, have accumulated in *S. nigrum*. This has led to variations in binding strength or ability of *AcMYB110* to the target DNA sequences of key enzymes, resulting in different anthocyanin compositions and, consequently, different colors in the two *S. nigrum* species. Recent research has indicated that only a few amino acid changes in the active site of the dihydroflavonoid 4-reductase (DFR) enzyme in petunias can transform the color of their flowers from pink to orange. This color shift is due to increased levels of pelargonidin [25]. In addition, distinct regulatory mechanisms, such as transcription factors or small RNA families, influence the activity of the *AcMYB110* transcription factor in two *S. nigrum* species. It is possible that these species interact differently with *AcMYB110* in the MBW transcription factors complex, which includes R2R3-MYB, bHLH, and WD-repeat. As a result, different compositions or quantities of anthocyanins are produced in transgenic *S. nigrum* species expressing *35S::AcMYB110*. The sequencing and resequencing of *S. americanum* genomes [26], sequencing of the *S. nigrum* genome [27], along with the establishment of a highly efficient genetic transformation system, provides a solid foundation for understanding the molecular mechanisms behind anthocyanin biosynthesis in these transgenic species.

## 5. Conclusions

A new, time-efficient genetic transformation method has been developed for the *S*. *nigrum*. The method enhances rooting, as demonstrated in tissue culture experiments using carnations. The novel method is expected to be broadly applicable to the genetic transformation and rapid propagation of various plants using tissue culture techniques.

## Data Availability

Data are contained within the article.
